# Outer Membrane Integrity-Dependent Fluorescence of the Japanese Eel UnaG Protein in Live *Escherichia coli* Cells

**DOI:** 10.3390/bios13020232

**Published:** 2023-02-07

**Authors:** Céline S. M. Richard, Hymonti Dey, Frode Øyen, Munazza Maqsood, Hans-Matti Blencke

**Affiliations:** The Norwegian College of Fishery Science, Faculty of Biosciences, Fisheries and Economics, UiT The Arctic University of Norway, N-9037 Tromsø, Norway

**Keywords:** reporter gene, synthetic biology, UnaG, outer membrane, bilirubin, biosensor

## Abstract

Reporter genes are important tools in many biological disciplines. The discovery of novel reporter genes is relatively rare. However, known reporter genes are constantly applied to novel applications. This study reports the performance of the bilirubin-dependent fluorescent protein UnaG from the Japanese eel *Anguilla japonicas* in live *Escherichia coli* cells in response to the disruption of outer membrane (OM) integrity at low bilirubin (BR) concentrations. Using the *E. coli* wild-type strain MC4100, its isogenic OM-deficient mutant strain NR698, and different OM-active compounds, we show that BR uptake and UnaG fluorescence depend on a leaky OM at concentrations of 10 µM BR and below, while fluorescence is mostly OM integrity-independent at concentrations above 50 µM BR. We suggest that these properties of the UnaG–BR couple might be applied as a biosensor as an alternative to the OM integrity assays currently in use.

## 1. Introduction

Reporter genes are important tools and are widely used in synthetic biology and cellular biosensors. Due to the ease of use and signal detection, fluorescent and bioluminescent reporter genes are utilized in different types of applications. They are usually fused to either a promoter–operator regulatory sequence or genes of interest and convert biological events into optically detectable signals, which can easily be read by appropriate instrumentation. The most common reporter genes currently in use are the green fluorescent protein *gfp* from the cnidarian *Aequorea victoria* [1,2,3] and the red fluorescent protein *rfp* from *Discosoma* coral [4,5,6], as well as different luciferases [7,8,9]. During the last decade, several alternatives to the traditional fluorescent proteins have emerged, most notably fluorescent proteins belonging to the fatty-acid-binding protein family, such as UnaG and SmurfP [10], as well as RNA-based light-up aptamers [11,12,13] such as spinach, broccoli, and pepper. They all have in common that they require a fluorogenic ligand for fluorescence, which often must be provided externally.

In this work, we tried to apply the fluorescent protein from a Japanese eel (*Anguilla japonica*), UnaG, for the ligand-dependent labeling of *Escherichia coli* cells [14]. This protein belongs to the fatty-acid-binding protein (FABP) family and produces fluorescence by binding to its ligand bilirubin (BR) (C_33_H_36_N_4_O_6_), a yellow–orange pigment. This molecule is an antioxidant tetrapyrrole, formed by the breakdown of heme—for example, from hemoglobin from dead red blood cells in the mammalian body. UnaG and the unconjugated BR bind noncovalently, but with high specificity and affinity. This protein has been successfully used as an imaging tool for live-cell fluorescence microscopy in mammalian cells [15,16,17], in yeast [18], as well as in bacteria for anaerobic imaging [19,20], or as a dark-to-green photoswitchable fluorescent protein for super-resolution imaging [17]. 

The necessity of ligands is not problematic if the host readily provides the molecules in sufficient quantities through its inherent metabolism. Therefore, the use of UnaG does not require the addition of BR in vertebrate systems. However, when UnaG is used as a reporter in bacteria, BR must be added externally, and, in the case of cytoplasmic expression of the protein, BR must pass the cell envelope and plasma membrane. As BR is a hydrophobic molecule with a size (584.7 Dalton) close to the exclusion threshold of outer membrane (OM) porins (ca. 600 Daltons) [21], sufficient access to BR inside the cell might be a limiting factor when expressed in Gram-negative bacteria. On the other hand, ligands, fluorophores, and enzyme substrate exclusion by cellular barriers such as the OM or plasma membrane can be used in assays or cellular biosensors to evaluate barrier integrity. This principle is used, for instance, in live–dead assays based on the fluorophores [22,23], where plasma membrane disruption is probed by propidium iodide access to the nucleic acids in the cytoplasm. Similarly, the exclusion of D-luciferin of intact plasma membranes is used in assays for plasma membrane integrity [24]. 

Several methods that are used to study the permeabilization of the OM of Gram-negative bacteria are based on similar principles—for instance, the use of the fluorescent probes 1-*N*-phenylnaphthylamine (NPN) [25,26,27,28,29] or 8-anilino-1-naphthylenesulfonic acid (ANS) [27,30], and ethidium bromide (EtBr) in a different assay [31], and spectrophotometric assays based on periplasmic β-lactamase activity and cytoplasmic β-galactosidase and the activation of respective enzyme-activated dyes [32]. In addition, GFP exported to the periplasm of Gram-negative bacteria has been used to assess damage to the OM in microscopy-based assays [33]. The latter principle has been further applied to multi-color fluorescent flowcytometry assays with GFP localized in the cytoplasm and mCherry in the periplasm [34,35]. The currently most widely applied approach seems to be the use of the fluorescent probe NPN. 

The toxicity of ligand, substrate, or probe to the cells of interest might negatively affect experiments or limit the usability for end point measurements rather than real-time assays. For example, a common assay for the evaluation of membrane potential in bacteria is based on the fluorescent dye DiOC2 [36]. The dye itself is cytotoxic and is therefore only used in end point assays. It has been found that also BR affects bacterial viability in the gut. A study by Nobles et al. [37] showed that BR can have a positive effect on the Gram-negative bacteria by protecting them from reactive oxygen species (ROS) but also a negative effect on Gram-positive bacteria by disrupting the plasma membrane at concentrations of at least 100 μM.

In this study, we show that UnaG-dependent fluorescence in living *E. coli* cells depends on OM disruption under the in vitro conditions that we tested when BR is added externally at relatively low concentrations.

## 2. Materials and Methods

### 2.1. Media and Growth Conditions

For cloning, *E. coli* strains were routinely grown in Luria–Bertani (LB) broth at 37 °C with aeration. For fluorescence measurements, the bacteria were grown in Mueller–Hinton (MH; Merck, Darmstad, Germany) broth medium at room temperature (RT) over-night, which was then diluted 1:100 in MH broth medium and grown to an OD_600_ of approximately 0.5 at RT. To avoid a fluorescence background, after centrifugation at 3000× *g* for 5 min, the bacteria pellet was washed by careful pipetting in 0.9% NaCl solution, 0.9% NaCl solution with 20 mM Tris HCl pH 7.5, phosphate-buffered saline (PBS), or 5 mM HEPES buffer (Sigma-Aldrich, St Louis, MO, USA), free of bilirubin (BR), before measuring fluorescence. Then, 1 mM BR (Sigma-Aldrich, St Louis, MO, USA) stocks were created in dimethyl sulfoxide (DMSO; Sigma-Aldrich, St Louis, MO, USA). Different concentrations of ampicillin (Merck KGaA, Darmstad, Germany), 100 μg/mL and 5 μg/mL, were used for the plasmid selection in *E. coli* MC4100 and NR698, respectively. 

### 2.2. Bacterial Strains and Plasmids

In this study, we used the isogenic *E. coli* K-12 strains MC4100 and NR698. To express UnaG constitutively in the cytoplasm, the plasmid pMM001 (Figure S11 and Sequence S1) was designed and then synthetized through Invitrogen GeneArt Gene Synthesis (Thermofisher, MA, USA) with codon optimization for *E. coli*. In this plasmid, the synthetic construct UnaG [14] is expressed from the constitutive OBX15 promoter [38]. The strain NR698 was constructed by Ruiz et al. [39], where the permeability of the OM increases by introducing the *imp4213* allele of *E. coli* BE100 [40] into the *E. coli* MC4100. This in-frame deletion of the *imp* gene, which encodes an essential protein of the OM assembly, results in a loss of OM integrity. For the membrane integrity assay, the strains were transformed with plasmid pCSS962 containing a constitutively expressed gene, *LucGR* [24].

### 2.3. Transformation

Competent *E. coli* MC4100 and NR698 were prepared by the transformation and storage solution (TSS) method [41]. Here, 100 μL of the competent strain was transformed with 100–500 ng plasmid. Cells were incubated at 37 °C, with agitation for 1 h, before being spread on LB agar plates with the appropriate antibiotics and incubated at 37 °C overnight.

### 2.4. Fluorescence Detection

A Synergy H1 Hybrid Reader (BioTek, Winooski, VT, USA) was used to measure the UnaG fluorescence of bacterial populations. To avoid excessive background fluorescence, the monochromator was set to an excitation wavelength of 508/8 nm and an emission wavelength of 538/8 nm, and fluorescence was measured in 30 s intervals at RT (the temperature inside the device was at 25.5 °C, slightly above ambient, throughout the measurements). The gain was kept at 100 in all experiments. Then, 90 μL of the bacterial suspension was added to a black round-bottom 96-well microtiter plate (Nunc, Roskilde, Denmark). BR and outer- and plasma-membrane-active compounds were added to the indicated concentrations. The following compounds were used: polymyxin B sulfate (PMB; Sigma-Aldrich, St Louis, MO, USA), polymyxin B nonapeptide (PMBN; GLPBIO, USA), chlorhexidine acetate (CHX; Fresenius Kabi, Halden, Norway). BR-free bacterial suspension served as the background, with water instead of PMB as a negative control. Data were processed with GraphPad Prism 9 software version 9.5.0 (GraphPad Software; Boston, MA, USA).

### 2.5. NPN Assays for Outer Membrane Integrity

The increased permeability of the OM was analyzed by measuring increased fluorescence as kinetics of 1-N-phenylnaphthylamine (NPN) uptake following the protocol described by Helander and Mattila-Sandholm [42]. Briefly, *E. coli* MC4100 and NR698 were grown overnight in MH broth medium. The cultures were further diluted and grown to OD_600_ 0.5, rinsed once using centrifugation at 3000× *g* for 5 min, and suspended in 5 mM HEPES buffer supplanted with 5 mM glucose (pH 7.2) and diluted to OD_600_ 0.5. NPN was added to a concentration of 20 µM containing 1 mL of cell suspension in HEPES buffer immediately prior to fluorescence monitoring using 96-well black-bottom microtiter plates. After 10 µL of the permeating agent was added to 90 µL of the cell suspension with NPN, fluorescence was measured using a microplate reader with excitation and emission wavelengths set to 350 and 420 nm, respectively. For the NPN OM assay with BR, *E. coli* cells were grown, harvested, and suspended as described above, before they were preincubated with different concentrations of BR for 10 min. BR-treated cells were washed once and resuspended in HEPES buffer, followed by the addition of NPN to obtain a final concentration of 20 µM for the measurement of fluorescence for 15 min. 

### 2.6. Luminescence Assays for Plasma Membrane Integrity

The *E. coli* strains MC4100 and NR698 constitutively expressing the luciferase LucGR from the pCSS692 plasmid were cultured overnight in MH broth medium supplemented with 5 μg/mL chloramphenicol (Merck KGaA, Darmstad, Germany) for *E. coli* NR698 and 20 μg/mL for MC4100. New day cultures were made by 1% inoculation in MH broth medium and incubated at RT with aeration until the OD_600_ reached 0.5. The final concentration of D-luciferin potassium salt (Synchem Inc., Elk Grove Village, IL, USA) in the medium was 2 mM. 

The real-time membrane integrity assay was modified from a previously described protocol for the membrane integrity assay [24]. This assay was performed on different strains of *E. coli*, including the wild-type (WT) MC4100 and the OM-deficient NR698 strains. The LucGR protein is dependent on its substrate D-luciferin to emit luminescence. PMB at a final concentration of 10 µg/mL was used as a positive control. Milli-H_2_O was used as a negative control. 

All values were normalized to the water control for the normalization of the luminescence. Data were processed with GraphPad Prism 9 software. 

### 2.7. Microscopy

Suspensions of UnaG-expressing *E. coli* strains MC4100 and NR698 in PBS buffer were prepared as described earlier. Sample preparation was identical as for the assays in the plate reader in PBS. First, 5 μL of bacterial suspension was transferred onto a microscopic slide and covered with a cover slip for immediate microscopic analysis. Fluorescence was analyzed at several time points after the addition of BR to 5 μM and PMB 10 µg/mL with a Leica DM6000B fluorescence microscope and an excitation light source, a Leica CTR6000, with the filter system Cube I3 DM 513828. Fluorescence was documented with a camera, a Leica DFC7000T, attached to the microscope. Identical camera settings were applied for all images taken. The imaging software used for image analysis was the Leica application suite LAS X, where identical settings for contrast enhancement were applied to the original micrographs. In addition, the brightness of the fluorescent images as a whole was increased to 150% in Photoshop CS6 version 13.0 (Adobe; San Jose, CA, USA) for better on-screen visibility. The original figure without enhanced brightness is supplied as Figure S12. 

## 3. Results and Discussion

### 3.1. UnaG Fluorescence in Complex Growth Media

**Figure 1:**
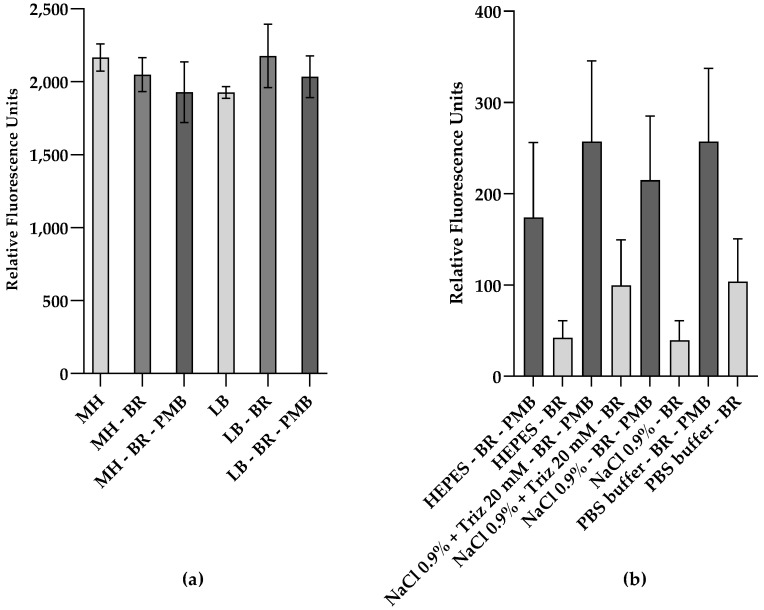
Relative fluorescence of UnaG in live *E. coli* cells is affected by the fluorescent background and the cell envelope active peptide polymyxin B (PMB). Relative fluorescence of *E. coli* MC4100 constitutively expressing UnaG from pMM001 after 15 min exposure to 5 μM bilirubin (BR) and 10 μg/mL of PMB in (**a**) MH medium or LB medium; (**b**) buffers and solutions, at an OD_600_ of 0.5. Relative fluorescence values are blanked to the respective control without BR. Each data point is the mean of three independent measurements.

To evaluate the fluorescence of UnaG expressed from a plasmid-based, constitutive promoter construct (pMM001) in the wild-type (WT) strain *E. coli* MC4100, the bacteria were grown in either MH or LB broth medium, two different complex media typically in use for different purposes and assays in our laboratory. Green fluorescence was analyzed after incubation in the presence of 5 µM BR. However, within the 15 min measurement window, no UnaG/BR-dependent increase in fluorescence was observed (Figure 1a). In addition, the background fluorescence of complex media is very high. It has been speculated that BR is excluded from entering bacterial cells by the wall of Gram-negative bacteria [43]. Therefore, we tried to compromise the OM with PMB, which is known to affect OM integrity [44]. Again, the addition of PMB did not result in an increase in UnaG-dependent fluorescence. 

### 3.2. The Effect of Different Buffers and Solutions on UnaG Fluorescence Signal-to-Noise Ratio after Membrane Disruption

To investigate whether the background fluorescence of complex media might camouflage any UnaG-dependent changes in fluorescence, we measured the fluorescence of the bacteria suspended in different buffers and solutions, which are often used with viable cells. Again, the WT strain *E. coli* MC4100 carrying the plasmid pMM001 was tested for UnaG-specific fluorescence. In addition, the influence of bacterial concentrations on signal-to-noise ratios was evaluated by testing the fluorescence of the bacterial suspension in HEPES buffer and in 0.9% NaCl solution at an OD_600_ of 0.1, 0.3, 0.5, 0.7, and 1.0. The signal-to-noise ratio seemed to increase with increasing cell concentrations until an OD_600_ of 0.5. Above this concentration, the fluorescence ratio of PMB+BR-treated cells to BR-treated cells stabilized (Figure S13). Hence, an OD_600_ of 0.5 was chosen for further experiments.

Figure 1b shows the comparison of the relative fluorescence emission of UnaG in the different buffer conditions at an OD_600_ of 0.5, after 15 min in the presence and absence of PMB at a final concentration of 10 μg/mL. The relative fluorescence was blanked to the background fluorescence in the absence of BR in each buffer/solution. Overall, the relative fluorescence of UnaG is strongest in cells treated with PMB when the cells are resuspended in PBS and HEPES, while the fluorescence increase in response to PMB treatment is most pronounced in 0.9% NaCl solution. In all tested conditions, the fluorescence increased at least two-fold after the addition of PMB, while the increase in 0.9% NaCl solution was approximately five-fold. The individual differences in UnaG fluorescence between the independent replicates resulted in a relatively high standard deviation. At the same time, the fold changes in all individual measurements were always largest when conducted in 0.9 % NaCl solution. Comparing the effect of PMB on fluorescence in complex media with corresponding data in buffers and solutions clearly indicated that the complex media affected the PMB-induced UnaG fluorescence (Figure 1a,b). Therefore, 0.9% NaCl solution was chosen for further experiments. This difference in fluorescence in response to PMB might be explained by the OM being impermeable to BR at these low concentrations. However, PMB also affects the membrane permeability of the plasma membrane, and, in our construct, UnaG was expressed and localized in the cytoplasm.

### 3.3. Outer-Membrane-Dependent Uptake of Bilirubin

**Figure 2:**
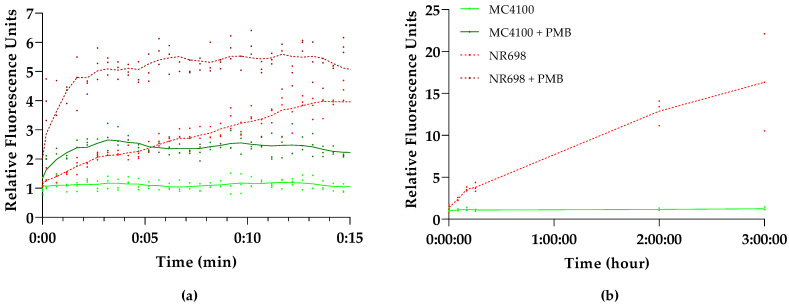
*E. coli* outer membrane (OM) integrity affects UnaG fluorescence. Kinetics of UnaG fluorescence in *E. coli* wild-type (WT) strain MC4100 (solid shades of green) and the isogenic *E. coli* OM-defective mutant NR698 (dashed shades of red) after (**a**) 15 min exposure of 10 μg/mL PMB and subsequent addition of 5 µM BR (dark shades) or 5 μM BR only (light shades); (**b**) 3 h exposure of 5 μM BR only. The data points represent three independent measurements normalized to the negative control of MC4100 in the presence of BR only. The mean is represented by the line of the same color.

To determine whether the fluorescence increase after the addition of PMB was caused by OM damage, the OM-deficient *E. coli* strain NR698 was transformed with pMM001. To study the effect of this OM deficiency on BR uptake, we compared the fluorescence kinetics of this mutant and its isogenic WT strain *E. coli* MC4100, both carrying the pMM001 plasmid. Figure 2a illustrates that the addition of BR alone immediately increases the fluorescence only in NR698, while the fluorescence of MC4100 remains at a constant low level. Within the 15 min measuring window used in this experiment, the fluorescence in the OM-deficient strain increases to four-fold compared to the WT strain. The addition of PMB, on the other hand, increases the relative fluorescence in the WT two-fold. Interestingly, the fluorescence of the OM-deficient strain also increases and stabilizes at approximately five-fold after the addition of PMB. This might be caused by the effect of PMB on the plasma membrane or additional damage to the OM. Moreover, when extending the measurement window to 3 h, the fluorescence of MC4100 stays at a low level, while the fluorescence of NR698 is constantly rising (Figure 2b). 

The results indicate that the OM indeed excludes BR from entering the cells as only the OM-compromised cells allow for the emission of fluorescence, either by mutation (NR698) or a permeabilizing agent (PMB). This seems to confirm the prediction by Chia et al. that the impermeability of bacterial cell walls to BR limits the use of UnaG to outer cell wall targets [43]. This might be caused by the size of BR as the exclusion threshold of OM porins is around 600 Daltons [21] and the molecular weight of BR is 584.7 Daltons. Possibly, BR is also actively removed from the cells with the help of efflux pumps. However, these experiments were conducted at relatively low concentrations of BR. At higher concentrations of BR, a rapid and concentration-dependent increase of fluorescence could be observed in the absence of compounds affecting OM integrity (see Figure S3 in Supplementary Materials), which coincides with the results from the original study in *E. coli* [14] and studies conducted in anaerobic conditions with different *Bacteroidetes* species [43] at 200 µM and 25 µM, respectively. We were not able to rule out or confirm that BR itself has an OM-permeabilizing effect at higher concentrations, as its absorbance spectrum interferes with the NPN-based fluorescence. To exclude any major damage to the OM by BR, we also conducted synergy studies incubating both strains in Minimum Inhibitory Concentration (MIC) assays with different combinations of BR and erythromycin or vancomycin. These antibiotics are efficiently excluded by the OM and therefore render Gram-negative bacteria relatively insensitive, compared to Gram-positive bacteria [45,46,47,48]. While the NR698 was sensitive to all antibiotic concentrations tested, MC4100 did not become more sensitive in the presence of BR. On the contrary, the highest BR concentrations seemed to reduce sensitivity at the MIC (see Table S1 in Supplementary Materials). This observation seems to be in accordance with BR protecting *E. coli* against oxidative stress, as shown in earlier studies [37]. 

### 3.4. Confirming Outer Membrane Integrity by NPN Assays 

**Figure 3:**
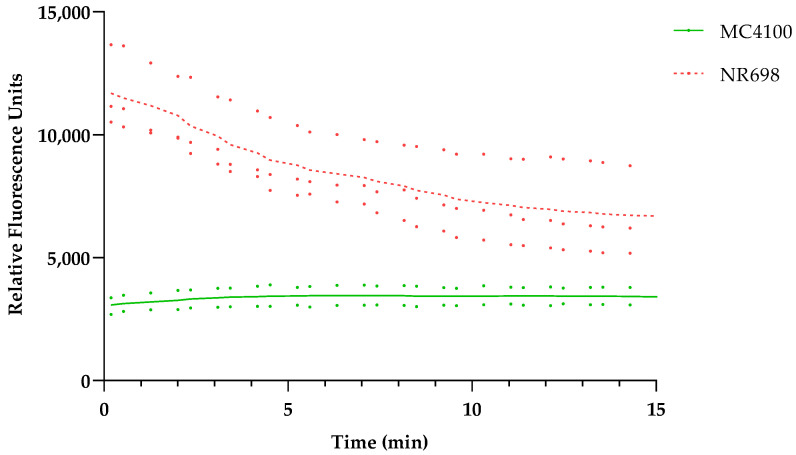
*E. coli* MC4100 and NR698 cells were used to detect fluorescence resulting from OM permeability to the small hydrophobic molecule 1-N-phenylnapthylamine (NPN). The data points represent two (MC4100, solid shade of green) or three (NR698, dashed shade of red) independent measurements normalized to the water-treated control (bacteria in 5 mM HEPES buffer). The mean is represented by the line of the same color.

To confirm the hypothesis that the OM is responsible for BR exclusion at low concentrations, we wanted to test the differences in OM integrity in both strains with the fluorescent probe 1-N-phenylnapthylamine (NPN), which is often used in OM integrity assays [25,26,27,28,29]. In addition, we confirmed the effects of PMB and EDTA on the OM of these *E. coli* strains. As mentioned above, PMB is known to disrupt the LPS layer of Gram-negative bacteria. NPN is a small hydrophobic molecule (219 Da) that cannot effectively cross the OM and fluoresces only weakly in aqueous solution but strongly when it is in close contact with phospholipid (PL) moieties, which become exposed in response to OM damage. When we compared the background fluorescence taking the same number of cells, the WT strain with intact OM (MC4100) produced weaker fluorescence than the strain with deficient OM (NR698). This difference in fluorescence decreases over time, since the fluorescence intensity of the OM-compromised strain decreases over time, as shown in Figure 3. It seems that NPN can easily access the periplasmic space and bind to the PL of the OM and outer leaflet of the inner membrane when the OM is compromised.

**Figure 4:**
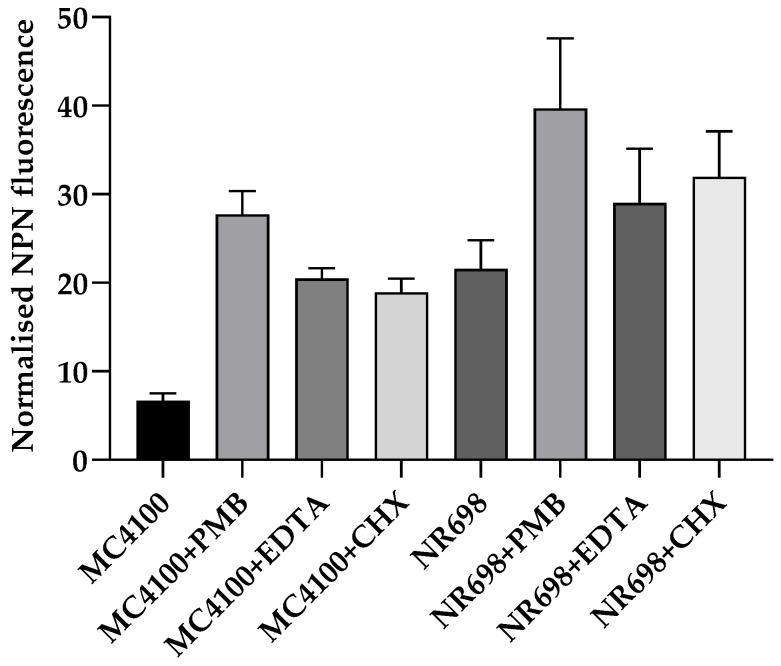
Relative NPN fluorescence in response to different membrane- and OM-active compounds. The permeability of the OM was assessed by measuring the fluorescence of NPN in both *E. coli* strains (MC4100 and NR698) after 2 min in the presence of 10 μg/mL PMB, 5 mM EDTA, or 100 μg/mL chlorhexidine (CHX). The fluorescence emission was plotted after normalizing all the samples to the bacteria in 5 mM HEPES buffer.

We then compared the effect of different OM-active compounds on the NPN fluorescence of both strains (Figure 4). MC4100 treated with 10 µg/mL PMB fluoresced almost six-fold more compared to the non-treated control, whereas CHX and EDTA showed a four-fold increase in fluorescence at the 2 min point, which is usually used for OM effects in the NPN assay [25,26]. It is worth noting that MC4100 became slightly more fluorescent in the presence of 10 µg/mL PMB and 5 mM EDTA than NR698 alone, even though a 1.5–2-fold increase in fluorescence was observed when NR698 cells were treated with 10 µg/mL PMB. However, MC4100 cells were less permeable when treated with 100 µg/mL CHX, which is known to be a strong membranolytic agent with an immediate effect on the viability of bacterial cells, although its OM activity at a sub-MIC level has not been established yet. In our assay, the higher fluorescence values in the NR698 strain with porous OM indicated that NR698 was already more permeable to NPN and reached its higher saturation level in the absence of membrane permeabilizers. 

### 3.5. Bilirubin Uptake Is Mostly Independent of Plasma Membrane Integrity

**Figure 5:**
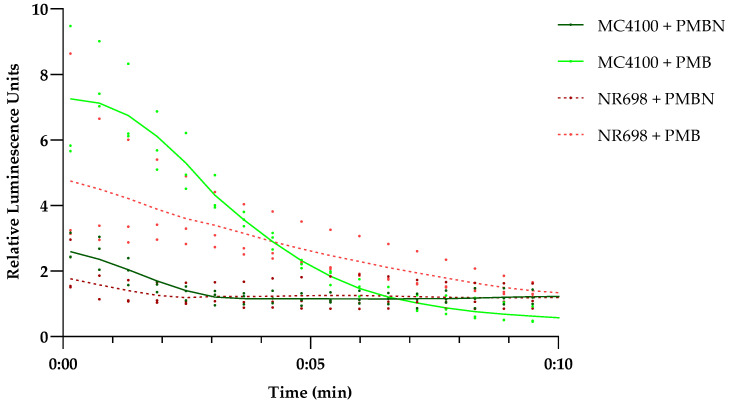
Effect of PMB and PMBN on plasma membrane integrity of *E. coli* MC4100 and NR698. Luminescence kinetics of LucGR in *E. coli* MC4100 (solid shades of green) and *E. coli* NR698 (dashed shades of red) in response to the presence of 12.5 µg/mL PMB (light color) or polymyxin B nonapeptide (PMBN) (dark color) and due to D-luciferin influx caused by plasma membrane permeabilization. The data points represent three independent measurements normalized to the negative control in the presence of D-luciferin only. The mean is represented by the solid line of the same color. An initial luminescence increase represents membrane permeabilization, the subsequent luminescence decrease represents ATP depletion due to bacterial cell death because of lost membrane integrity, while luminescence stabilization on the level of the control indicates survival of the main population with limited or no plasma membrane damage.

To evaluate the plasma membrane integrity of the mutant strain NR698 and the isogenic WT MC4100, we transformed both strains with plasmid pCSS962 coding for a constitutively expressed eukaryotic luciferase LucGR. This construct was used to evaluate the integrity of the plasma membrane [24]. In addition, we wanted to confirm that a derivative of PMB, the PMB nonapeptide (PMBN), did not affect the plasma membrane of either strain at 10 µg/mL, as we planned to use it as an example for a substance specifically damaging the OM. This peptide is described to be highly specific for the efficient perturbation of the OM and affects the plasma membrane only at high concentrations [49]. The integrity of the plasma membrane of *E. coli* MC4100 and NR698 (Figure 5) was tested in response to PMB and PMBN at a concentration of 12.5 μg/mL, and the kinetics of the bioluminescence of the protein LucGR was measured for 10 min after the addition of each compound. The luminescence increased directly after the addition of PMB in both strains. This indicates that the plasma membrane is compromised, allowing D-luciferin to diffuse into the cells and the enzyme to emit luminescence. PMBN, on the other hand, did affect luminescence to a lower extent in either strain at the tested concentration. Furthermore, the luminescence stabilized to a level similar to the non-treated control, confirming that plasma membrane integrity is not severely perturbed by PMBN in either *E. coli* strain and that the plasma membrane of the mutant NR698 in the absence of antimicrobial compounds is still excluding D-luciferin. As BR itself has earlier been described to affect membrane stability [37,50,51,52], we also analyzed how different concentrations of BR affect plasma membrane integrity. Although the highest concentration of BR resulted in a two-fold increase in luminescence in MC4100, the luminescence levels did not reflect the same pattern as known for membrane-active compounds (see Figures S4 and S5). We also tested different concentrations of BR against an *E. coli* viability sensor based on the *lux* operon, without observing a concentration-dependent specific effect apart from partial light absorption by bilirubin (see Figures S7 and S8). This is also in agreement with MIC studies conducted earlier, where BR, even at the highest concentrations tested, did not inhibit growth [53], and which we have confirmed in our lab for both strains used in this study. 

### 3.6. Is UnaG a Suitable Sensor for Outer Membrane Damage?

**Figure 6:**
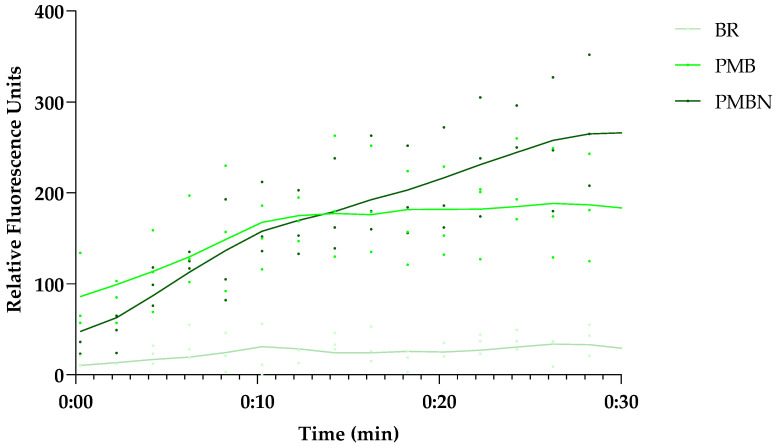
Both the OM-active PMBN and PMB induce UnaG fluorescence in the presence of BR, but show differences in the kinetics over time. The 30 min fluorescence kinetics of UnaG in *E. coli* MC4100 after exposure to 10 µg/mL PMB and 5 µM BR (medium green), 10 µg/mL PMBN and 5 µM BR (dark green), or 5 µM BR only (light green). The data points represent three independent measurements normalized to the negative control in the presence of bacteria only. The mean is represented by the solid line of the same color.

To evaluate whether UnaG-expressing *E. coli* strains could be used as indicators of OM damage, the effect of PMBN on UnaG fluorescence, and therefore BR diffusion through the OM, was tested. *E. coli* MC4100 was subjected to PMB and PMBN carrying the plasmid pMM001 with the constitutively expressed UnaG gene. Figure 6 shows the kinetics of the fluorescence increase after the addition of 10 µg/mL of either peptide. It is evident that both peptides substantially increase the fluorescence. As we showed earlier that the PMBN does not seem to have a major effect on plasma membrane integrity at the tested concentration, this effect is specific for OM damage. However, as studies with *Bacteroidetes* [19] grown anaerobically have shown that BR is taken up by the cells when provided with the growth medium over time, use of the UnaG–BR combination might require strict control of the assay conditions. Although there are already several different assays to test OM damage, in some cases, there might be advantages of using UnaG in combination with BR. Interestingly, the long-term stability of the system over several hours (Figure 2b) suggests a possible application of the system in assays with living bacterial biosensors for the longer-term monitoring of OM integrity in real time. 

**Figure 7:**
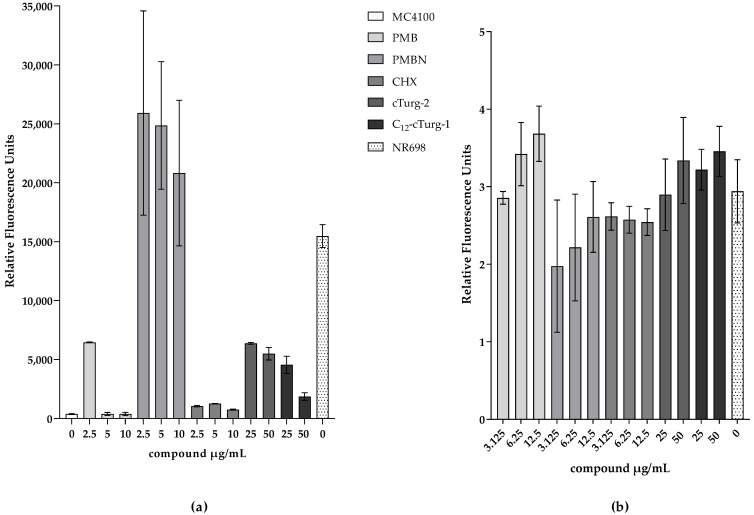
Comparison of relative fluorescence readouts of the proposed UnaG-based OM integrity biosensor and the traditional NPN assay in response to membrane damage. Representative fluorescence values from three independent measurements are shown. (**a**) Fluorescence of MC4100 constitutively expressing UnaG from pMM001 in 0.9% NaCl 10 h after addition of 5 μM BR and indicated concentrations of OM- or plasma-membrane-active compounds. The measurements were blanked to the respective control without BR. (**b**) Normalized fluorescence in presence of NPN and different concentrations of OM- or plasma-membrane-active compounds in 5 mM HEPES after 3 min to the MC4100 control with NPN and only water.

**Figure 8:**
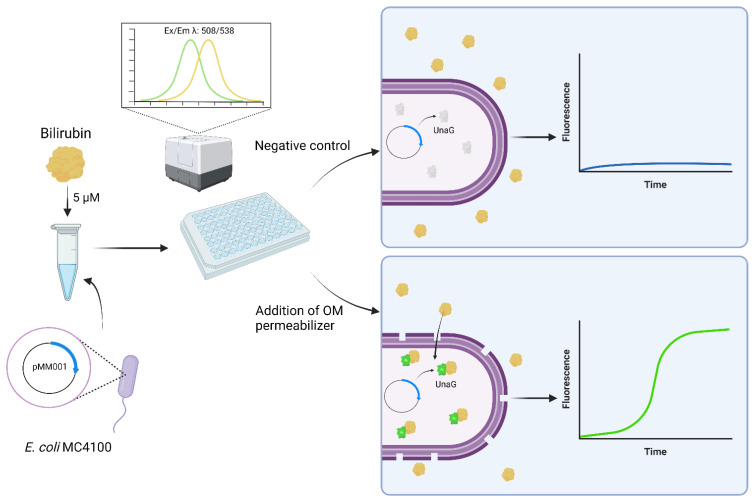
Schematic of the use of the reporter protein UnaG as an OM permeabilization whole-cell biosensor. Created with BioRender.com (accessed on 6 December 2022).

To test this hypothesis, the fluorescence of *E. coli* MC4100 carrying the plasmid pMM001 was measured for 10 h in the presence of different concentrations of compounds that are known or suspected to show OM-disrupting activity, plasma-membrane-disruptive activity, or both. PMB has been described to affect the integrity of Gram-negative membranes [44,54], while its derivative PMBN seems to permeabilize the OM down to concentrations around 1 µg/mL [55] and it does not seem to affect plasma membrane activity at 12.5 µg/mL (compare Figure 5); hence, its permeabilizing activity is likely exclusively affecting the OM at concentrations between 1 and 10 µM. Here, we show that PMB induces UnaG fluorescence only at the lowest concentration tested (2.5 µg/mL), while PMBN-induced fluorescence is up to three-fold higher at all concentrations tested, including 10 µM. The fluorescence intensities of bacteria treated with PMBN resemble the fluorescence intensity of the OM mutant NR698. The concentration-dependent fluorescence of PMB might be explained by its bacteriostatic effect at low concentrations and its bactericidal effect at high concentrations [54]. Chlorhexidine (CHX), on the other hand, only slightly induces fluorescence at the tested concentrations, while both PMB and CHX permeabilize the plasma membrane at the higher concentrations. This possibly indicates that CHX attacks the OM to a lesser extent, with the plasma membrane being the main target. In addition, we tested two recently described cyclic antimicrobial peptide (Turgencins) derivatives, the peptide analogue cTurg-2 as well as the lipopeptide analogue C_12_-cTurg-1 [56], against the prospective UnaG-based biosensor. Again, the fluorescence levels in response to the analytes varied. C_12_-cTurg-1, which was previously described to disrupt both the plasma membrane and OM, causes an increase in UnaG fluorescence at both concentrations tested, while the observed fluorescence is more than two-fold stronger at the lower concentration. cTurg-2, on the other hand, was described as mostly OM-active and, in its presence, UnaG fluorescence rose to above the level of the lower concentration of C_12_-cTurg-1 at both concentrations tested (Figure 7). This might indicate that cTurg-2 is indeed mostly active against the OM. NPN assays conducted for chlorhexidine published previously [56], and related unpublished data on PMB and the Turgencin derivatives summarized in Figure 7b, all show increasing fluorescence with increasing concentrations of active analytes. The NPN assay seems to quantify the combined membrane damage of both the OM and the plasma membrane. Therefore, the fluorescence intensity tends to increase with increasing analyte concentrations as opposed to the UnaG-based fluorescence, which decreases with increasing analyte concentrations. It is tempting to speculate that plasma-membrane-active compounds kill the bacterial cells due to plasma membrane permeabilization at and above the MIC. Loss of viability shuts down all cellular metabolism, including protein/UnaG synthesis. On the other hand, OM-active compounds such as PMBN will not damage the plasma membrane and cells stay alive, constantly expressing UnaG, with BR diffusing through the compromised OM as it is bound to the protein in the cytoplasm. The steady fluorescence increases over 10 h is represented in the kinetic fluorescence curves shown in Figures S9 and S10 for all the compounds tested. Therefore, we propose that this sensor construct could be used in assays to identify outer membrane active compounds as illustrated in Figure 8.

### 3.7. Is There a Variation in the OM Effect within the Population?

**Figure 9:**
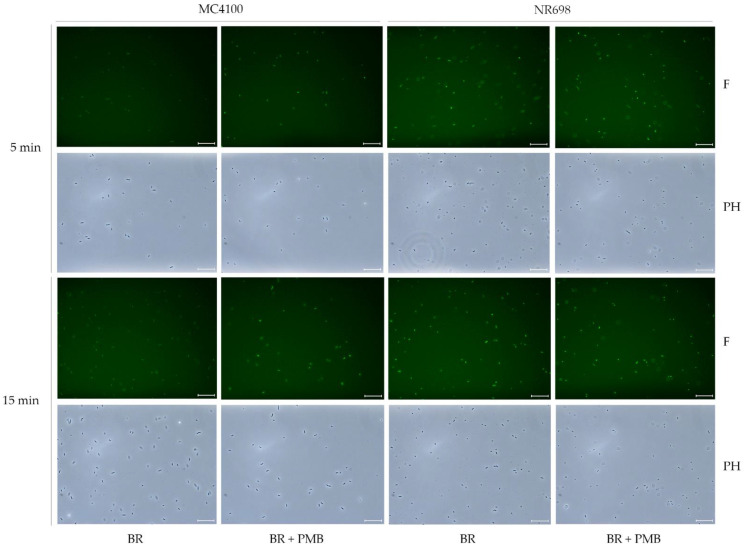
PMB induces population-wide fluorescence of UnaG-expressing *E. coli* cells. Fluorescence images of *E. coli* MC4100 and NR698 at time points of 5 min and 15 min after exposure to BR (5 μM) (BR) or BR and PMB (10 μg/mL) (BR + PMB) at x400 magnification. The images were taken with the phase contrast (PH) and with fluorescence (F) through the software LAS X. The scale bars represent 25 μm. For better visibility on all monitors, the brightness of the fluorescent pictures (F) was equivalently increased to 150 with Adobe Photoshop CS6 version 13.0.

To rule out the possibility that a minor subpopulation is responsible for the increase in fluorescence in response to treatment with OM-disrupting agents observed by the experiments in the plate reader, samples treated in a similar fashion with PMB were analyzed under a fluorescence microscope. Several fluorescent microscope images of the different suspensions of UnaG-expressing *E. coli* strains MC4100 and NR698 in PBS were taken at two different time points after exposure to PMB at 10 μg/mL and BR at 5 μM or BR only (Figure 9). The bacteria were planktonic, viable, and freely moving in the buffer. Therefore, the exposure time could not be increased to achieve brighter images as the moving bacteria resulted in blurry images; this is also visible as a slight positional change between phase contrast and fluorescent images. The fluorescence of the WT MC4100 after exposure to BR was significantly lower compared to the fluorescence after exposure to BR and PMB. Moreover, fluorescence seemed to be mostly constant throughout the population in the focal plane. The effect of PMB was detectable 5 min after its addition. As expected, the fluorescence was significantly lower in the WT compared to the OM-deficient strain when exposed to BR only. This confirms the results observed with PMB performed in the plate reader and indicates that the observed fluorescence is due to the relatively equal fluorescence of the whole population, rather than strongly fluorescent subpopulations. However, an earlier study used the protein UnaG for the imaging of anaerobic bacteria without any OM-disrupting agent [19]. In their study, the concentration of BR in the medium was 25 μM—a concentration only five-times higher than used in this study. Therefore, higher concentrations of BR might increase the diffusion rate to an extent, where sufficient BR molecules accumulate inside the cytoplasm to induce fluorescence also in the absence of OM permeabilizers. It is important to note that this microscopic study was conducted before deciding on the use of 0.9 % NaCl solution as the medium of choice for conducting experiments, and it was conducted in PBS. More importantly, in the microscopic study, PMB was used as the OM-permeabilizing agent at 10 µg/mL. We later showed, in microtiter plate assays, that the addition of PMBN or PMB at 2.5 µg/mL resulted in substantially higher fluorescence emission than PMB at 10 µg/mL, indicating a more OM-specific effect (Figure 7 and Figure S9). However, in the presence of BR, the untreated OM-compromised NR698 strain emitted strong fluorescence, which was independent of any compromising agents used, and it can therefore serve as a benchmark to compare fluorescence between the assays conducted in the plate reader and the microscopic observations. In conclusion, the microscopic images show that the fluorescence throughout the treated and untreated populations seems to be mostly homogenous and confirms that a breach in OM integrity is necessary for strong fluorescence. 

## 4. Conclusions

UnaG fluorescence in *E. coli* is completely dependent on the external addition of its ligand BR. At concentrations of 50 µg/mL and above, the presence of BR alone ensures the sufficient diffusion of BR through the cell envelope for maximal fluorescence in live cells. At low concentrations of BR (5 µg/mL), diffusion and subsequent fluorescence is dependent on OM disruption. Furthermore, BR does not seem to affect plasma membrane integrity or the survival of *E. coli* cells negatively. Therefore, the UnaG–BR couple might be used as a real-time reporter system in OM integrity biosensors, especially in cases where non-immediate activity and/or OM-specific activity needs to be detected over an extended period, beyond the 2 min mostly used for NPN-based assays. Due to the relatively simple setup, the system might be used as a biosensor that can distinguish OM from OM- and plasma-membrane-active compounds in high-throughput screening applications.

## Data Availability

Not applicable.

## Supplementary Materials

The following supporting information can be downloaded at: https://www.mdpi.com/article/10.3390/bios13020232/s1, Figure S1: NPN kinetics in response to different permeabilizing analytes; Figure S2: The dose-dependent short-term effect of PMB and PMBN on UnaG fluorescence kinetics; Figure S3: Fluorescence kinetics of UnaG of *E. coli* MC4100 after exposure of different concentrations of BR; Figure S4: Luminescence kinetics of LucGR in *E. coli* NR698 after exposure of different concentrations of BR; Figure S5: Luminescence kinetics of LucGR in *E. coli* MC4100 after exposure of different concentrations of BR; Figure S6: Plasma membrane remains intact after exposure to PMBN; Figure S7: No effect of BR concentrations on short-term viability of *E. coli* MC4100; Figure S8: *E. coli* NR698 stays alive after exposure to different BR concentrations; Figure S9: Long-term fluorescence kinetics of the proposed OM biosensor to well-known model peptides; Figure S10: Long-term fluorescence kinetics of the proposed OM biosensor to novel cyclic peptide derivatives; Figure S11: Map of the plasmid pMM001 (Benchling.com; accessed on 2 December 2022); Figure S12: PMB induces population-wide fluorescence of UnaG-expressing *E. coli* cells; Figure S13: The influence of bacterial density on UnaG fluorescence; Table S1: Antimicrobial activity (MIC in μg/mL); Sequence S1: DNA sequence of the plasmid pMM001.
