# Supplementary material for: Outer Membrane Integrity-Dependent Fluorescence of the Japanese Eel UnaG Protein in Live Escherichia coli Cells
Source: Biosensors (Basel). 2023 Feb 7;13(2):232. doi: 10.3390/bios13020232 (PMC9953992; doi:10.3390/bios13020232)
Supplement: Supplementary file 1 [file biosensors-13-00232-s001.zip › biosensors-2119843-supplementary.pdf]

biosensors

Supporting Information

Outer Membrane Integrity-Dependent Fluorescence of the Japanese Eel UnaG Protein in Live *Escherichia coli* Cells

Céline S. M. Richard, Hymonti Dey, Frode Øyen, Munazza Maqsood and Hans-Matti Blencke

Methods

Minimum inhibitory concentration (MIC) assay

Stock solutions of Bilirubin and further dilutions were prepared by dissolving them in 100% DMSO. The final DMSO concentration remained 2% in all the concentration of each antibiotic, Bilirubin or DMSO alone. A modified broth microdilution susceptibility assay, based on the CLSI M07-A9 protocol, was used to determine minimal inhibitory concentrations (MIC) [1]. Briefly, overnight bacterial cultures were grown in Mueller-Hinton (MH) medium (Difco Laboratories, USA) for 2 hours at room temperature. The bacterial inoculum was diluted to 5 x 10^5 cells/mL in MH medium and added in 96-well plates (Nunc, Roskilde, Denmark) preloaded with two-fold dilution series of Bilirubin (200 to 1.6 μM) and antibiotic solutions (64 to 0.5 μg/mL) in a ratio of 1:10 giving a final well volume of 100 μL with bacterial inoculum. The microplates were incubated in an EnVision 2103 microplate reader (PerkinElmer, Llantrisant, UK) at 35 °C, with OD_{595} recorded every hour for 24 h. The minimal inhibitory concentration (MIC) value was defined the lowest concentration of antibiotics either in presence or absence of Bilirubin showing an optical density less than 10% of the negative (growth) control, consisting of bacteria and MQ- water.

Bacterial viability assay (luminescence based)

The *E. coli* strains MC4100 and NR698 were transformed with plasmid pCGLS-11 [2] expressing a *luxCDABE* operon from a constitutive promoter. Both strains were cultured overnight in MH broth medium supplemented with 100 and 5 μg/mL Ampicillin (Merck KGaA, Darmstad, Germany), respectively. New day cultures were made by 1% inoculation in MH broth medium and incubated at RT with aeration until the OD600 reached 0.5. This was changed from earlier use of the viability assay to adjust the cell density to the UnaG assays. To evaluate the effect of Bilirubin on bacterial viability the luminescence values were normalized to the DMSO control to account for DMSO related increase of luminescence. Data were processed with GraphPad Prism 9 software.

Figures and tables

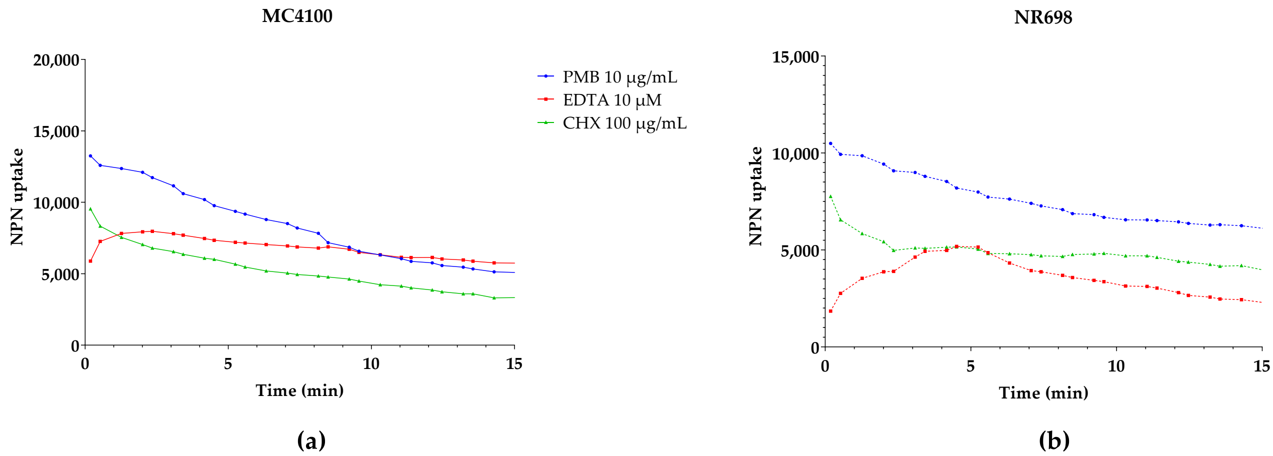

Figure S1. NPN kinetics in response to different permeabilizing analytes. *E. coli* MC4100 (**a**) and NR698 (**b**) cells were used to detect fluorescence as a result of outer membrane (OM) permeabilization to the small hydrophobic molecule 1-N-phenylnapthylamine (NPN). Fluorescence emission normalized to the water treated control (bacteria in HEPES buffer) is plotted as NPN uptake over time (min).

Table S1. Antimicrobial activity (MIC in µg/mL)

	MIC (µg/mL)	
**Antibiotic**	**MC4100**	**NR698**
Vancomycin in 2% DMSO	64	0.25 – 0.4
Vancomycin + BR (2% DMSO)	>64	>0.4
Erythromycin 2% DMSO	16	0.25 – 0.4
Erythromycin + BR (2% DMSO)	32	>0.8

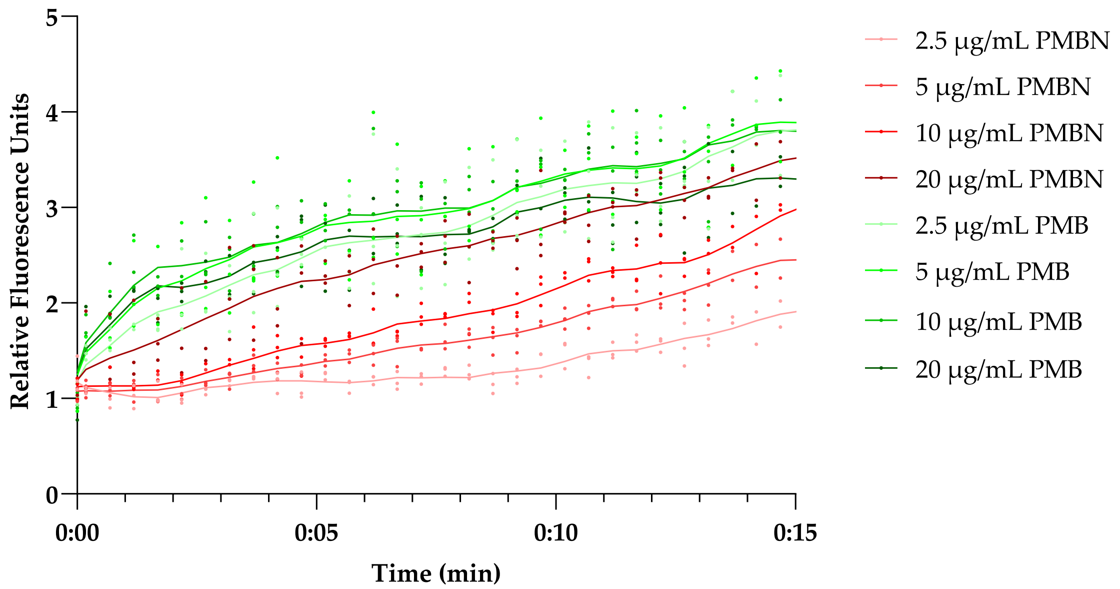

Figure S2. The dose dependent short-term effect of PMB and PMBN on UnaG fluorescence kinetics. Fluorescence kinetic of UnaG of *E. coli* MC4100 after exposure of different concentrations of polymyxin B (PMB; solid shades of green) or polymyxin B nonapeptide (PMBN; solid shades of red).

The data points represent three independent measurements normalized to the negative control in presence of BR only. The mean is represented by the solid line of the same color.

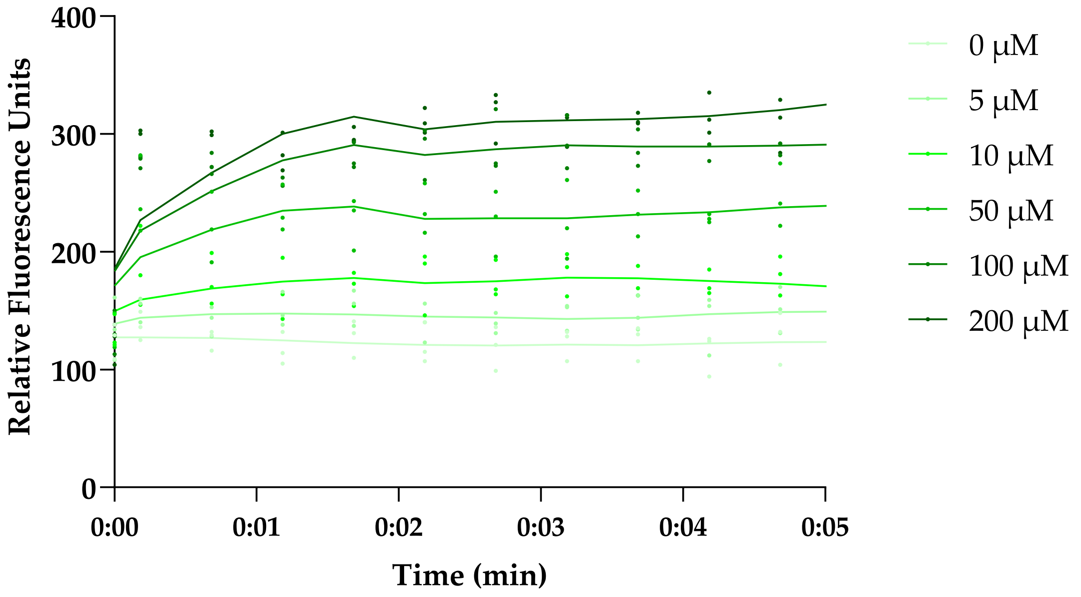

Figure S3. Fluorescence kinetic of UnaG of *E. coli* MC4100 after exposure of different concentration of BR. The data points represent three independent measurements normalized to the negative control in presence of bacteria only. The mean is represented by the solid line of the same color.

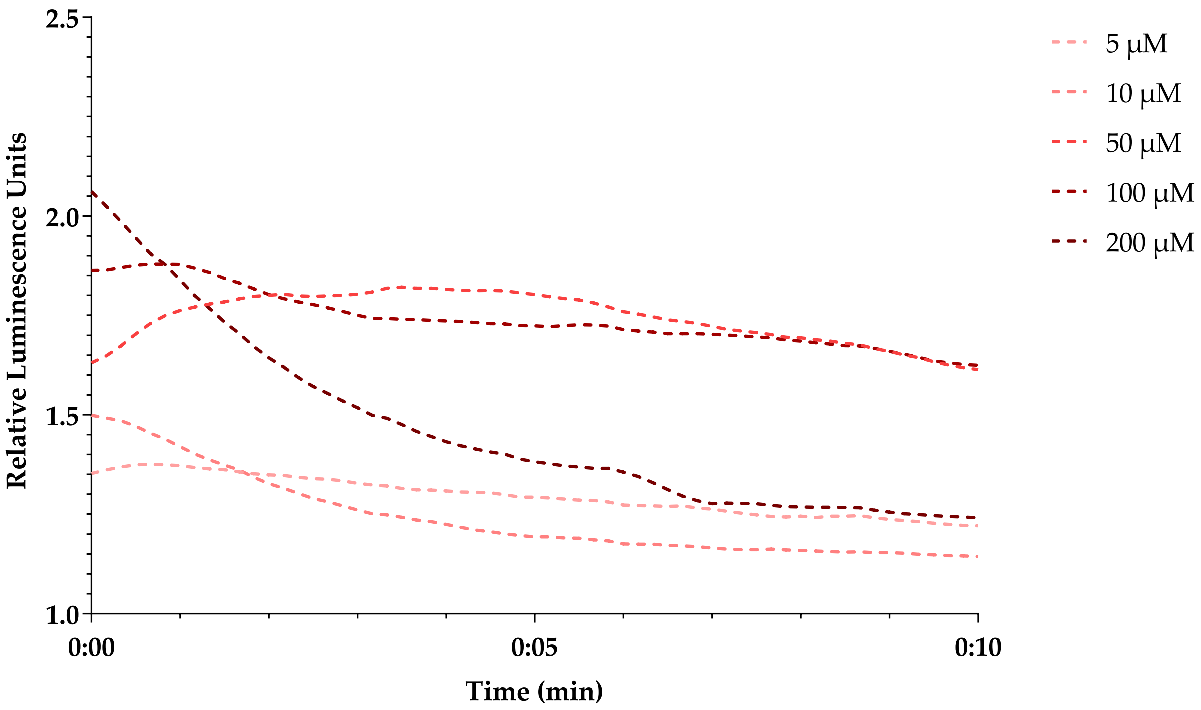

Figure S4. Luminescence kinetic of LucGR in *E. coli* NR698 after exposure of different concentration of BR. The mean of three independent measurements normalized to the negative control in presence of D-luciferin only is represented by the dashed line of the same color.

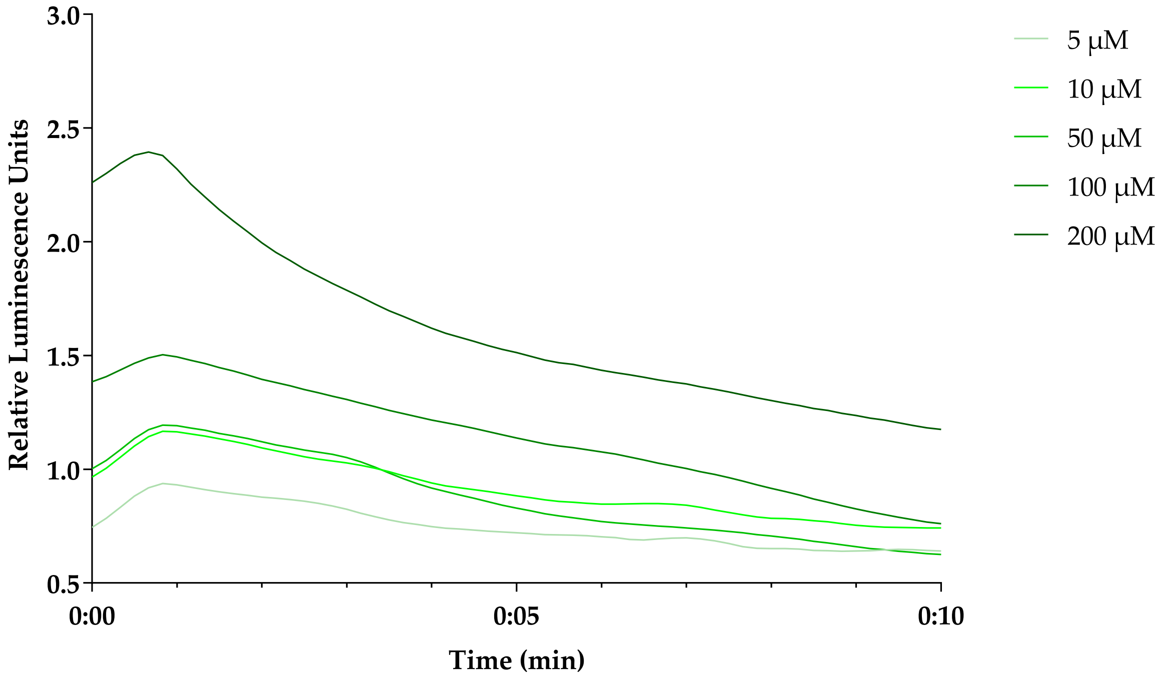

Figure S5. Luminescence kinetic of LucGR in *E. coli* MC4100 after exposure of different concentration of BR. The mean of three independent measurements normalized to the negative control in presence of D-luciferin only is represented by the solid line of the same color.

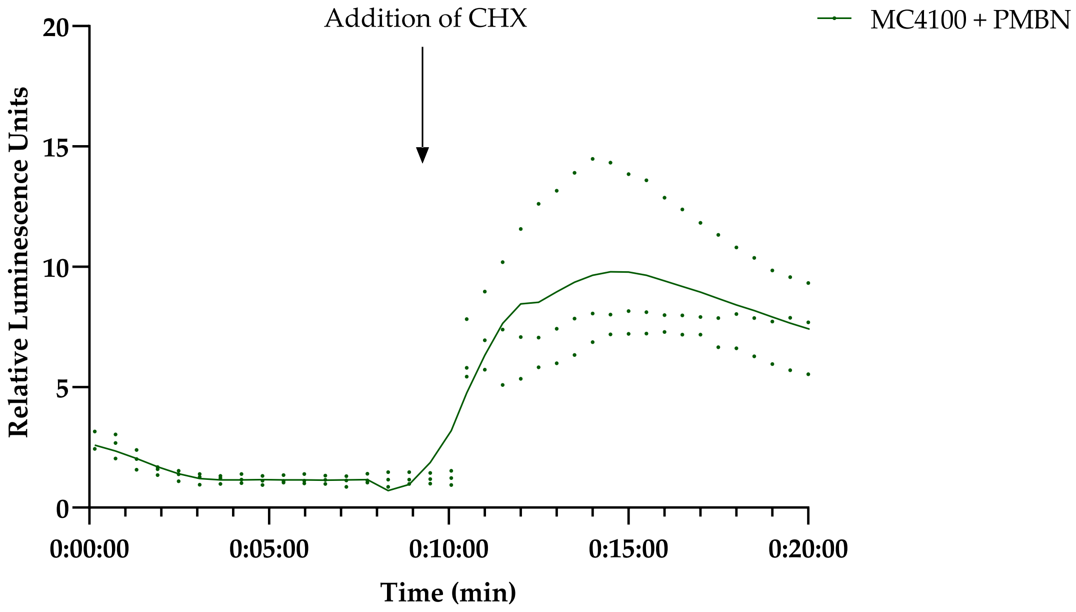

Figure S6. Plasma membrane remains intact after exposure to PMBN. Luminescence kinetic of LucGR in *E. coli* MC4100 after initial exposure to 12,5 µg/mL PMBN and 5 µM of BR and subsequent addition of 5 µg/mL chlorhexidine (CHX) at the 10 minutes mark. The data points represent three independent measurements normalized to the negative control in presence of D-luciferin only. The mean is represented by the solid line of the same color.

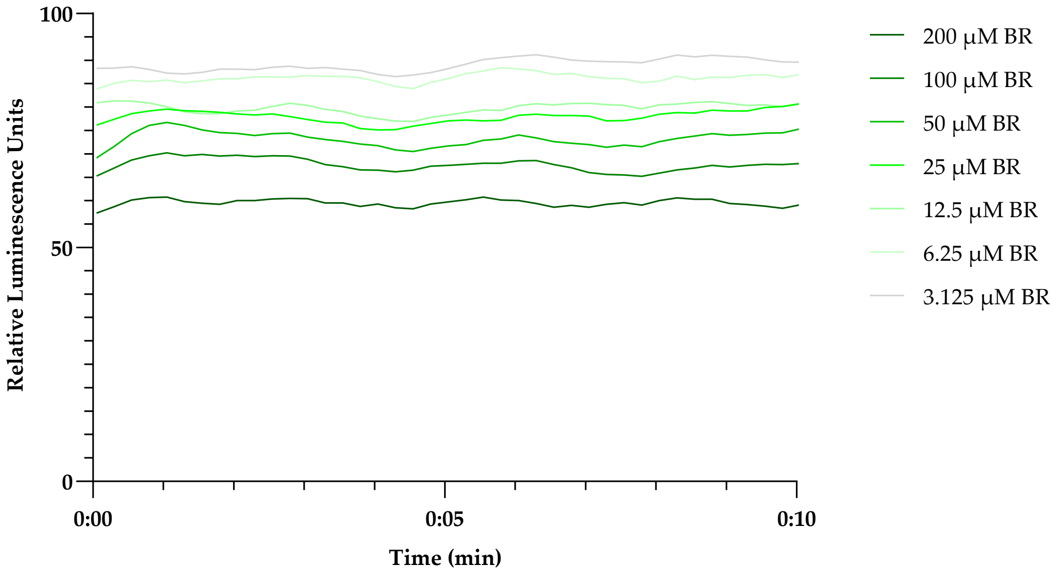

Figure S7. No effect of BR concentrations on short-term viability of *E. coli* MC4100. Luminescence kinetic of lux operon in *E. coli* MC4100 after exposure to different concentrations of BR. The mean of three independent measurements normalized to the negative control in presence of DMSO only is represented by the solid lines. Concentration dependent reduction of luminescence is likely caused by the absorbance spectrum of BR overlapping with the emission spectrum of the luciferase. A decrease in luminescence over time would indicate reduced viability.

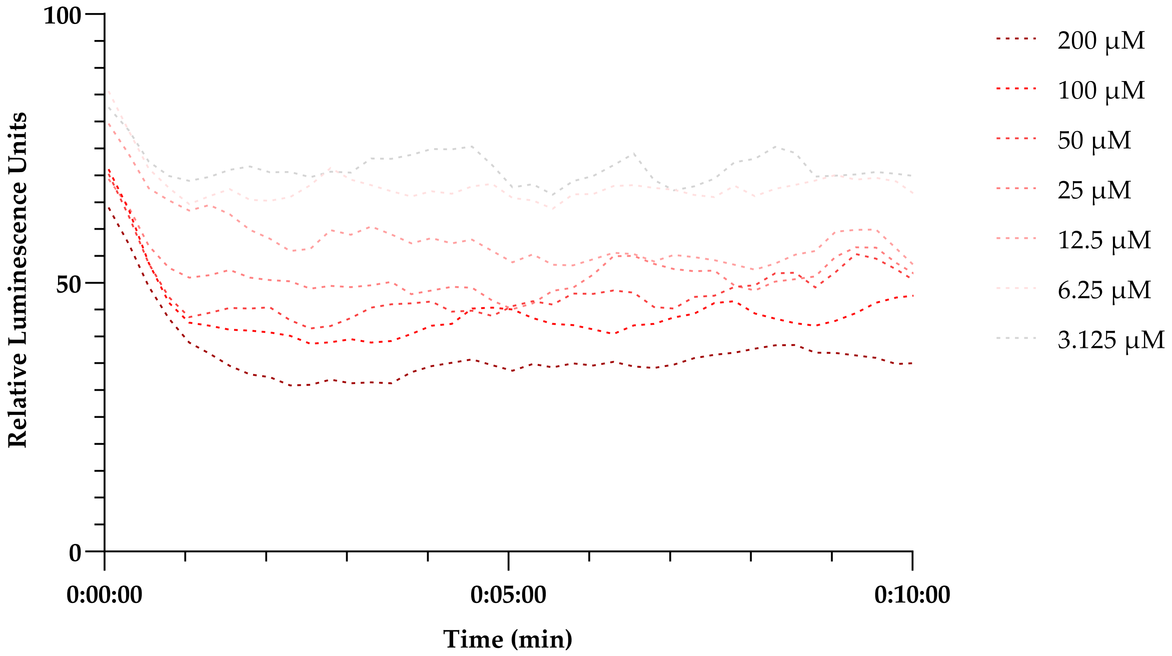

Figure S8. *E. coli* NR698 stays alive after exposure to different BR concentrations. Luminescence kinetic of the lux operon in *E. coli* NR698 after exposure of different concentration of BR. The mean of three independent measurements normalized to the negative control in presence of DMSO only is represented by the dashed line of the same color. Concentration dependent reduction of luminescence is likely caused by the absorbance spectrum of BR overlapping with the emission spectrum of the luciferase.

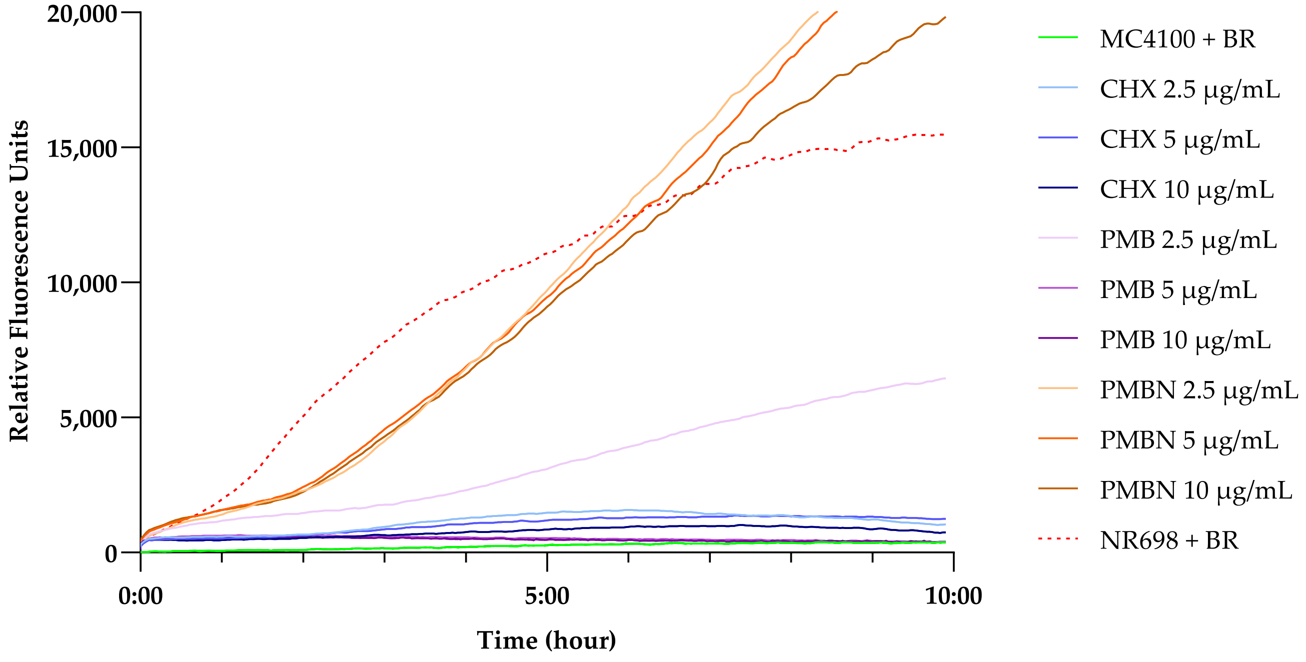

Figure S9. Long-term fluorescence kinetic of the proposed OM biosensor to well-known model peptides. Fluorescence kinetic of UnaG of *E. coli* MC4100 after exposure to different concentrations of PMB (solid shades of purple), PMBN (solid shades of orange), and CHX (solid shades of blue) for 10 hours. The *E. coli* MC4100 negative control with bilirubin only is represented by a solid green line and *E. coli* NR698 with bilirubin only by a dashed red line. All the data are normalized to bacteria with no addition of bilirubin. The mean of three independent measurements is represented by the solid line of the same color.

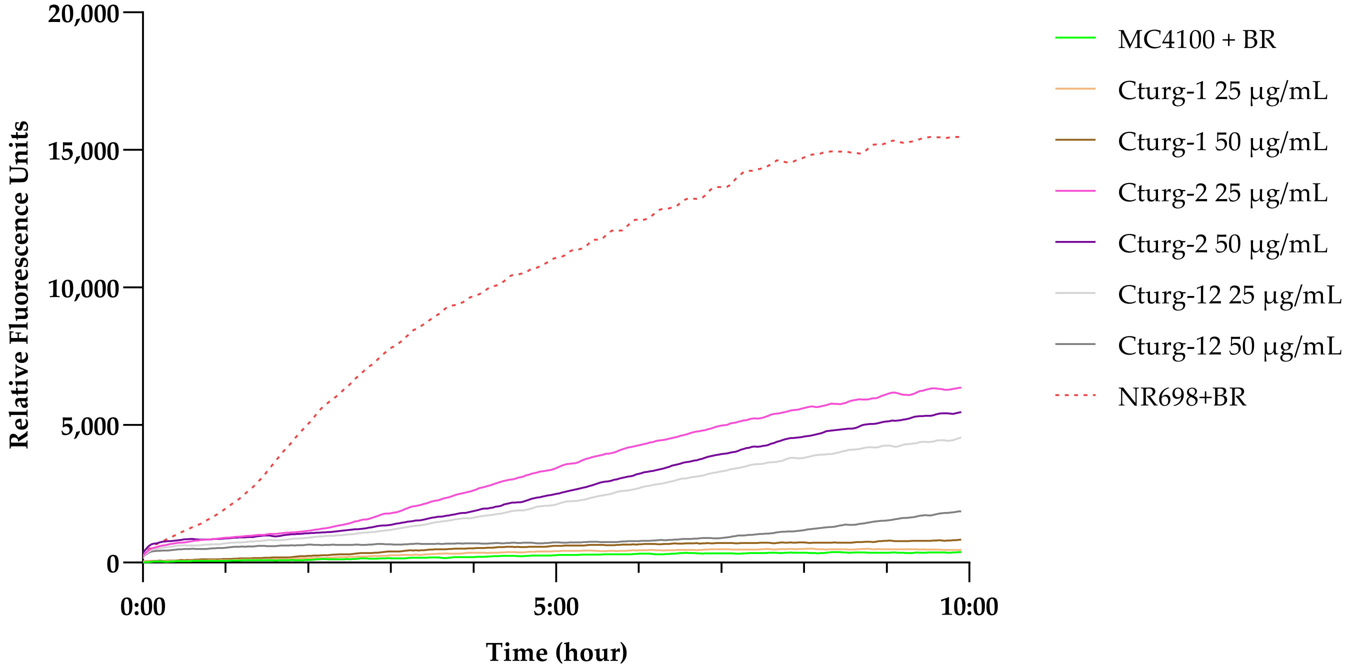

Figure S10. Long term fluorescence kinetic of the proposed OM biosensor to novel cyclic peptide derivatives. Fluorescence kinetic of UnaG in *E. coli* MC4100 after exposure to different concentrations of cyclic marine antimicrobial peptide derivatives cTurg-1 (solid shades of brown), cTurg-2

(solid shades of pink), and derivate C_{12}-Turg-1 (solid shades of blue) for 10 hours. The control of *E. coli* MC4100 with bilirubin only is represented by a solid green line and *E. coli* NR698 with bilirubin only by a dashed red line. All the data are normalized to bacteria with no addition of bilirubin. The mean of three independent measurements is represented by the solid line of the same color.

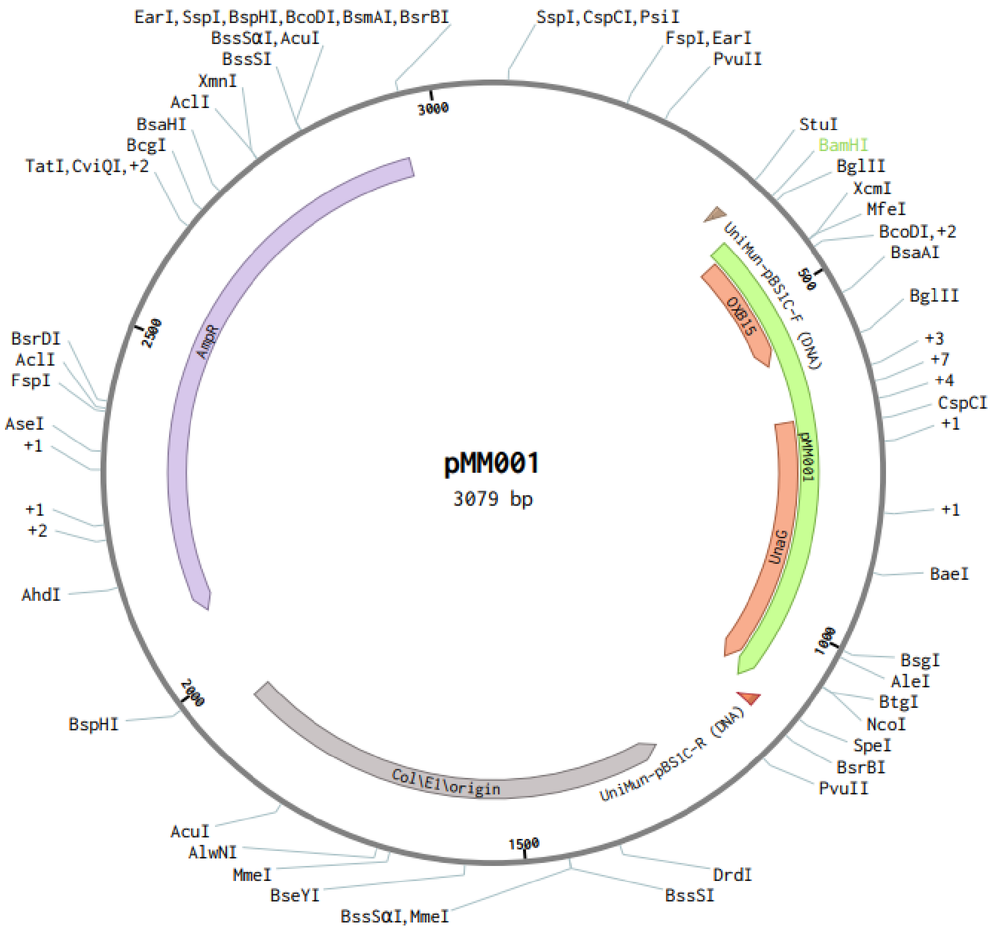

Figure S11. Map of the plasmid pMM001 (Benchling.com; accessed on 2 December 2022).

Sequence S1. DNA sequence of the plasmid pMM001.

CTAAATTGTAAGCGTTAATATTTTGTTAAAATTCGCGTTAAATTTTTGTTAAATCA
GCTCATTTTTTAACCAATAGGCCGAAATCGGCAAAATCCCTTATAAATCAAAAG
AATAGACCGAGATAGGGTTGAGTGGCCGCTACAGGGCGCTCCCATTCGCCATTC
AGGCTGCGCAACTGTTGGGAAGGGCGTTTCGGTGCGGGCCTCTTCGCTATTACGC
CAGCTGGCGAAAGGGGGATGTGCTGCAAGGCGATTAAGTTGGGTAACGCCAGG
GTTTTCCCAGTCACGACGTTGTAAAACGACGGCCAGTGAGCGCGACGTAATACG
ACTCACTATAGGGCGAAGGAAGGCCGTCAAGGCCTTGGCGGAAGGCCGTCAAG
GCCGCATGGATCCCCAGATCTAAGCTGTTGTGACCGCTTGCTCTAGCCAGCTATC
GAGTTGTGAACCGATCCATCTAGCAATTGGTCTCGATCTAGCGATAGGCTTCGAT
CTAGCTATGTAGAAACGCCGTGTGCTCGATCGCTTGATAAGGTCCACGTAGCTGC
TATAGTTGCTTCAACAGAACATATTGACTATCCGGTATTACCCGGCAGATCTTTG
TCGATCCTACCATCCACTCGACACACCCGCCAGCGGCCGCTGCCAAGCTTCCGA
GCTCTCGAATTCAAAGGAGGTACCCACCATGGTTGAAAAATTTGTTGGCACCTG
GAAAATTGCCGATAGCCATAATTTTGGCGAATACCTGAAAGCCATTGGTGCACC
GAAAGAACTGAGTGATGGTGGTGATGCAACCACACCGACACTGTATATTAGCCA
GAAAGATGGTGATAAGATGACCGTGAAAATTGAAAATGGTCCGCCTACCTTTCT
GGATACCCAGGTTAAATTCAAACTGGGCGAAGAATTTGATGAATTTCCGAGCGA
TCGTCGTAAAGGTGTTAAAAGCGTTGTTAATCTGGTGGGTGAAAAACTGGTTTAT
GTGCAGAAATGGGATGGTAAAGAAACCACCTATGTGCGCGAAATCAAAGATGG
TAAACTGGTTGTTACCCTGACCATGGGTGATGTTGTTGCAGTTCGTAGCTATCGTC
GTGCAACCGAATAAACTAGTCTGGGCCTCATGGGCCTTCCGCTCACTGCCCGCTT
TCCAGTCGGGAAACCTGTCGTGCCAGCTGCATTAACATGGTCATAGCTGTTTCCT

TGCGTATTGGGCGCTCTCCGCTTCCTCGCTCACTGACTCGCTGCGCTCGGTCGTTC
GGGTAAAGCCTGGGGTGCCTAATGAGCAAAAGGCCAGCAAAAGGCCAGGAAC
CGTAAAAAGGCCGCGTTGCTGGCGTTTTTCCATAGGCTCCGCCCCCCTGACGAGC
ATCACAAAAATCGACGCTCAAGTCAGAGGTGGCGAAACCCGACAGGACTATAA
AGATACCAGGCGTTTCCCCCTGGAAGCTCCCTCGTGCGCTCTCCTGTTCCGACCC
TGCCGCTTACCGGATACCTGTCCGCCTTTCTCCCTTCGGGAAGCGTGGCGCTTTCT
CATAGCTCACGCTGTAGGTATCTCAGTTCGGTGTAGGTCGTTCGCTCCAAGCTGG
GCTGTGTGCACGAACCCCCCGTTCAGCCCGACCGCTGCGCCTTATCCGGTAACTA
TCGTCTTGAGTCCAACCCGGTAAGACACGACTTATCGCCACTGGCAGCAGCCAC
TGGTAACAGGATTAGCAGAGCGAGGTATGTAGGCGGTGCTACAGAGTTCTTGAA
GTGGTGGCCTAACTACGGCTACACTAGAAGAACAGTATTTGGTATCTGCGCTCTG
CTGAAGCCAGTTACCTTCGGAAAAAGAGTTGGTAGCTCTTGATCCGGCAAACAA
ACCACCGCTGGTAGCGGTGGTTTTTTTGTTTGCAAGCAGCAGATTACGCGCAGAA
AAAAAGGATCTCAAGAAGATCCTTTGATCTTTTCTACGGGGTCTGACGCTCAGTG
GAACGAAAACTCACGTTAAGGGATTTTGGTCATGAGATTATCAAAAAGGATCTT
CACCTAGATCCTTTTAAATTAAAAATGAAGTTTTAAATCAATCTAAAGTATATAT
GAGTAAACTTGGTCTGACAGTTACCAATGCTTAATCAGTGAGGCACCTATCTCA
GCGATCTGTCTATTTCGTTCATCCATAGTTGCCTGACTCCCCGTCGTGTAGATAAC
TACGATACGGGAGGGCTTACCATCTGGCCCCAGTGCTGCAATGATACCGCGAGA
ACCACGCTCACCGGCTCCAGATTTATCAGCAATAAACCAGCCAGCCGGAAGGG
CCGAGCGCAGAAGTGGTCCTGCAACTTTATCCGCCTCCATCCAGTCTATTAATTG
TTGCCGGGAAGCTAGAGTAAGTAGTTCGCCAGTTAATAGTTTGCGCAACGTTGTT
GCCATTGCTACAGGCATCGTGGTGTCACGCTCGTCGTTTGGTATGGCTTCATTCA
GCTCCGGTTCCCAACGATCAAGGCGAGTTACATGATCCCCCATGTTGTGCAAAA
AAGCGGTTAGCTCCTTCGGTCCTCCGATCGTTGTCAGAAGTAAGTTGGCCGCAGT
GTTATCACTCATGGTTATGGCAGCACTGCATAATTCTCTTACTGTCATGCCATCCG
TAAGATGCTTTTCTGTGACTGGTGAGTACTCAACCAAGTCATTCTGAGAATAGTG
TATGCGGCGACCGAGTTGCTCTTGCCCGGCGTCAATACGGGATAATACCGCGCC
ACATAGCAGAACTTTAAAAGTGCTCATCATTGGAAAACGTTCTTCGGGGCGAAA
ACTCTCAAGGATCTTACCGCTGTTGAGATCCAGTTCGATGTAACCCACTCGTGCA
CCCAACTGATCTTCAGCATCTTTTACTTTCACCAGCGTTTCTGGGTGAGCAAAAA
CAGGAAGGCAAAATGCCGCAAAAAAGGGAATAAGGGCGACACGGAAATGTTG
AATACTCATACTCTTCCTTTTTCAATATTATTGAAGCATTTATCAGGGTTATTGTCT
CATGAGCGGATACATATTTGAATGTATTTAGAAAAATAAACAAATAGGGGTTCC
GCGCACATTTCCCCGAAAAGTGCCAC

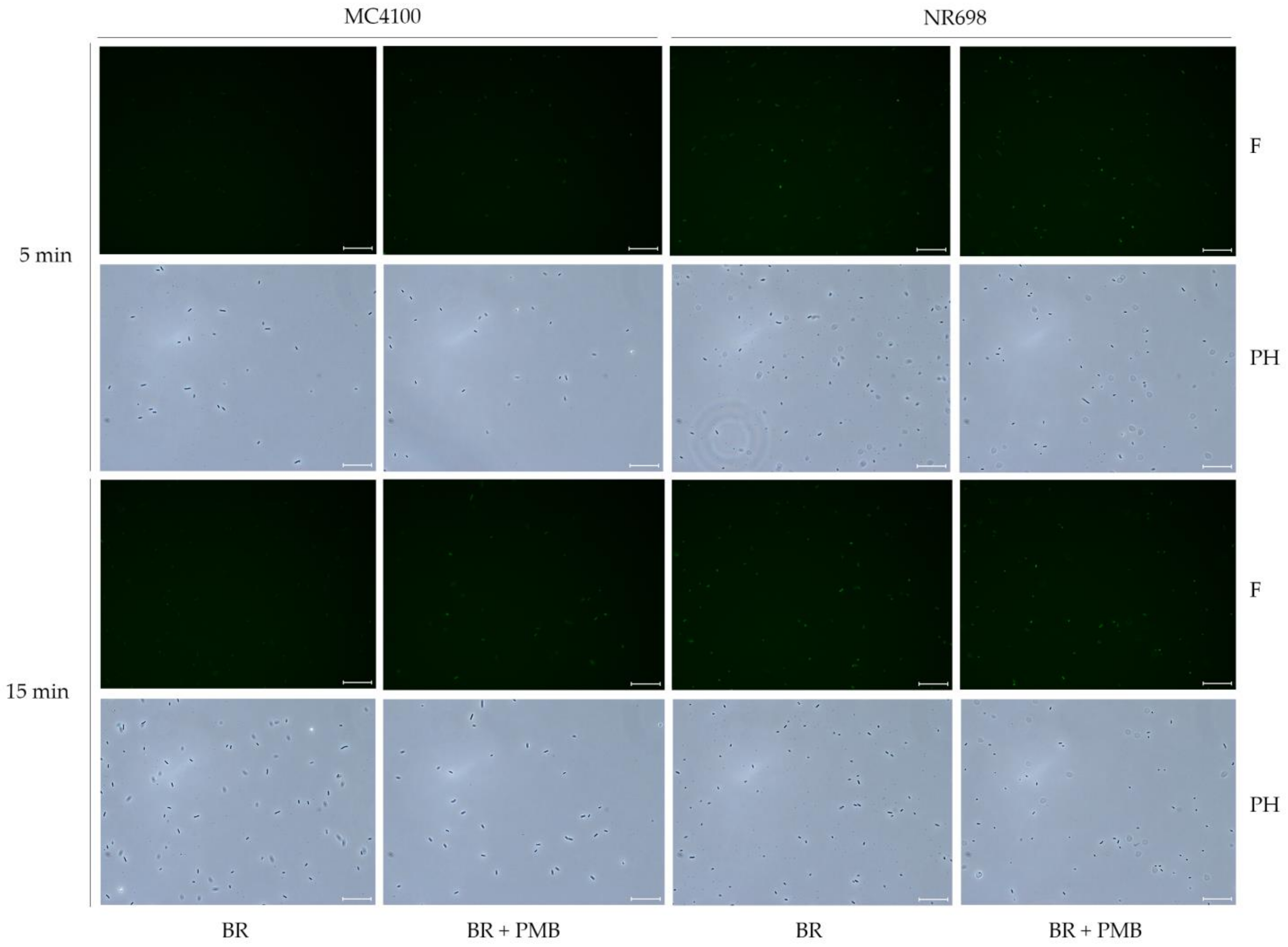

Figure S12. PMB induces population wide fluorescence of UnaG expressing *E. coli* cells. Fluorescence images of *E. coli* MC4100 and NR698 at time points of 5 minutes and 15 minutes after exposure to BR (5 μM) (BR) or BR and PMB (10 μg/mL) (BR + PMB) at x400 magnification. The images were taken with the phase contrast (PH) and with fluorescence (F) through the software LAS X. The scale bars represent 25 μm. Version without the increase of the brightness through Adobe Photoshop CS6 version 13.0.

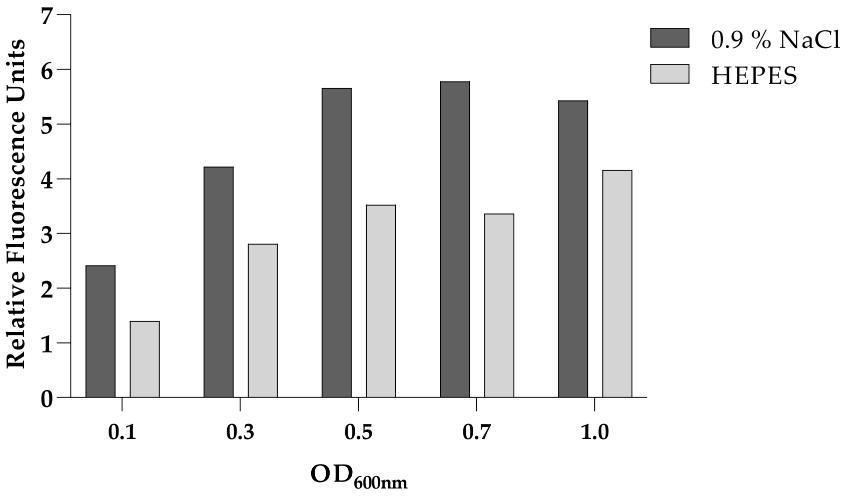

Figure S13. The influence of bacterial density on UnaG fluorescence. Relative fluorescence of UnaG in E. coli MC4100 after 15 min exposure to 10 µg/mL PMB normalised to the control in absence of PMB at different ODs in 0.9% NaCl and HEPES buffer
